# Folic acid supplementation in children with sickle cell disease: a randomized double-blind noninferiority cross-over trial

**DOI:** 10.1016/j.ajcnut.2025.02.001

**Published:** 2025-02-05

**Authors:** Brock A Williams, Heather McCartney, Joel Singer, Angela M Devlin, Suzanne Vercauteren, Ali Amid, John K Wu, Crystal D Karakochuk

**Affiliations:** 1 Food, Nutrition, and Health Program, Faculty of Land and Food Systems, The University of British Columbia, Vancouver, British Columbia, Canada; 2 BC Children’s Hospital Research Institute, Vancouver, British Columbia, Canada; 3 Department of Pediatrics, Faculty of Medicine, the University of British Columbia, Vancouver, British Columbia, Canada; 4 School of Population and Public Health, Faculty of Medicine, The University of British Columbia, Vancouver, British Columbia, Canada; 5 The Centre for Health Evaluation and Outcome Science, Vancouver, British Columbia, Canada; 6 Department of Pathology and Laboratory Medicine, BC Children’s Hospital, Vancouver, British Columbia, Canada

**Keywords:** folic acid, micronutrient supplementation, one carbon metabolism, sickle cell disease, pediatrics, randomized control trial

## Abstract

**Background:**

Children with sickle cell disease (SCD) in Canada are routinely supplemented with folic acid to provide sufficient folate for the increased demands of erythropoiesis. However, with the mandatory folic acid fortification of refined grains and pharmacotherapies that extend the lifespan of sickled red blood cells (RBC), this clinical practice is in question.

**Objectives:**

This study aims to determine the efficacy of folic acid supplementation by measuring the effect of 12 ± 1 wk of 1 mg/d folic acid, compared with placebo, on concentrations of RBC folate (primary outcome), serum folate, and 1-carbon-related metabolites, and clinical outcomes in children with SCD.

**Methods:**

In this double-blind randomized controlled noninferiority cross-over trial, 31 children with SCD, aged 2–19 y, were enrolled and randomly assigned (1:1 with blocks of 4) to 1 mg/d folic acid, the current standard of care, or a placebo for 12 ± 1 wk. After a 12 ± 1 wk washout period, treatments were reversed.

**Results:**

The mean [95% confidence interval (CI)] difference in endline RBC folate concentrations across treatments was –179 (–260, –99) nmol/L, with the lower boundary of the CI exceeding noninferiority but the upper boundary not (*P* = 0.0001; modified intention-to-treat). There was no significant difference in the number of participants who had RBC folate deficiency after each treatment (*P* = 0.059). No participants presented with serum folate deficiency (<7 nmol/L). There were no significant differences observed in 1-carbon metabolite concentrations (total homocysteine, *S*-adenosylhomocysteine, *S*-adenosylmethionine, vitamin B_12_, or methylmalonic acid), hematological measures, nor clinical outcomes (specifically acute pain episodes or megaloblastic changes) when individuals were supplemented with folic acid in comparison with placebo.

**Conclusions:**

Despite mandatory food fortification and advances in the medical treatment of SCD, it appears that some children with this condition may still benefit from daily folic acid supplementation. Whether this translates to improved clinical outcomes remains uncertain.

This trial was registered at clinicaltrials.gov as NCT04011345 (https://clinicaltrials.gov/study/NCT04011345).

## Introduction

Sickle cell disease (SCD) is an autosomal recessive genetic disorder that results in structural alterations of the β-subunit of hemoglobin [1,2]. Chronic hemolysis and consequential increases in erythropoiesis in SCD are thought to increase the requirements of folate, a water-soluble family of compounds that play an essential role in erythropoiesis [3,4]. It is common clinical practice to recommend supplementation of 1–5 mg/d of folic acid, a synthetic form of folate, for individuals with SCD in Canada [5]. However, even just 1 mg/d folic acid exceeds the tolerable upper intake level for children aged 1–18 y [6], and intakes of folic acid as low as ∼0.2 mg have been shown to result in the elevation of unmetabolized folic acid (UMFA) concentrations in circulation [7,8]. The biological effects of UMFA are not fully understood; however, a National Institutes of Health Workshop (2020) on understanding the metabolic and clinical effects of excess folates/folic acid identified an emerging need for research to determine whether UMFA produces any adverse effects and the associated biological pathways [9].

In individuals with SCD, there is evidence that folic acid supplementation increases serum folate concentrations, but limited evidence that it improves hematological or clinical outcomes [3]. The only trial eligible for inclusion in the most recent Cochrane Review of folate supplementation in SCD was a 1983 trial among 117 Jamaican children with SCD aged 6 mo to 4 y supplemented with either 5 mg/d folic acid or a placebo [10]. Although the authors observed an increase in serum folate levels after 6 mo to 1 y of treatment with folic acid (concentrations <11 nmol/L were evident in 15% of children in the placebo group compared with none of the folic acid group), no effect on hemoglobin, growth parameters, or other clinical events was observed [10].

There is some limited evidence that children with SCD in North America can maintain sufficient folate status in the era of mandatory food folic acid fortification and widespread use of hydroxyurea, a medication that significantly extends the mean lifespan of RBCs in individuals with SCD through the promotion of fetal hemoglobin [11]. Nguyen et al. demonstrated that among children with SCD (*n* = 72) in the United States, the majority (96%) of whom were prescribed hydroxyurea, mean RBC folate concentrations were not significantly different in those who had 1 mg/d folic acid discontinued for >80 d compared with <80 d [12]. However, only 6 children had paired measurements of RBC folate concentrations pre- and post-discontinuation of supplementation and there was a high degree of variability [12].

As no well-designed randomized controlled trial of folic acid supplementation on children with SCD has been conducted to date in the postfolic acid fortification era, the aim of this study was to assess the efficacy of folic acid supplementation by measuring the effect of 12 ± 1 wk of 1 mg/d folic acid, compared with placebo, on concentrations of RBC folate (primary outcome), serum folate, and 1-carbon-related metabolites [total homocysteine, *S*-adenosylhomocysteine (SAH), *S*-adenosylmethionine (SAM), vitamin B_12_, and methylmalonic acid (MMA)], and clinical outcomes (megaloblastic changes and acute pain crisis frequency and severity) in children with SCD.

## Methods

### Study design

This was a double-blind randomized controlled cross-over trial of oral folic acid or placebo supplements for 12 ± 1 wk, followed by a 12 ± 1 wk washout period and a subsequent reversal of treatments. The study was conducted in Vancouver, Canada. Participant screening and recruitment started in February 2020 and was paused because of COVID-19 pandemic restrictions on research activities until October 2020. The first participant was enrolled in November 2020 and the last participant was enrolled in February 2022. Study enrolment was closed in March 2022, 9 mo in advance of study supplement expiry in November 2022 (to allow completion of all study interventions). At this point, all eligible individuals at the site had been approached regarding participation in the study. The trial was completed on 31 October, 2022.

Ethics approval was obtained from the University of British Columbia Clinical Research Ethics Board (H18-02981) and a Notice of Authorization was obtained for this clinical trial from the Natural and Non-Prescription Health Products Directorate of Health Canada. Written informed consent was obtained from all guardians and assent was obtained from children and adolescents, as applicable. The trial was registered with clinicaltrials.gov (NCT04011345), and the study protocol has been published [13].

### Participants

The study enrolled participants who met all the following inclusion criteria: a confirmed diagnosis of SCD, aged 2–19 y and attending BC Children’s Hospital for medical care and having reported receiving routine daily supplementation of folic acid for the prior ≥12 wk. Individuals were excluded from participation if they received a blood transfusion in the previous 12 wk, were allergic to any components of the placebo or folic acid supplements, presented with megaloblastic anemia in the previous 12 wk, had current pulmonary, renal and/or cardiac complications (for example, severe or recurrent acute chest syndrome), routinely took medications known to interfere with B-vitamin metabolism (chloramphenicol, methotrexate, metformin, sulfasalazine, phenobarbital, primidone, triamterene, and barbiturates), were currently pregnant, planning to become pregnant in the next 9 mo, or currently breastfeeding, had participated in a clinical research trial in the previous 30 d, donated blood in the previous 30 d, or had an unstable medical condition or laboratory results.

### Randomization and masking

Folic acid and placebo supplements were manufactured by Natural Factors Nutritional Products Ltd. (Coquitlam) as opaque capsules identical in appearance and bottle packaging except for a sticker that identified the treatment group (A or B). The folic acid and placebo supplements were analyzed for content at the start of the trial via high-performance liquid chromatography (HPLC) after manufacture [14]. An independent research assistant (Natural Factors Nutritional Products Ltd.) assigned blinding codes (A or B) to the folic acid and placebo supplements. The allocation sequence to treatment A or B was determined by our clinical trialist via computer-generated random numbers in SAS statistical software, stratified in blocks of 4 to ensure roughly equal distribution of treatment order allocations. The generated allocation sequence and blinding codes were transferred directly via encrypted email to the research pharmacy (BC Children’s Hospital) who dispensed the supplements to maintain blinding of allocation. Clinicians/care providers, study personnel, outcome assessors, data analysts, and participants were blinded to the treatment allocation sequence. The treatment allocation was kept confidential by research pharmacy personnel until data analysis of the primary outcome (RBC folate concentrations) was completed.

### Procedures

All participants had been on routine prophylactic folic acid supplementation of 1 mg/d (clinical standard) for ≥12 wk before enrolment in the trial. Participants were instructed to discontinue routine folic acid supplements the day before the start of study interventions. Enrolled participants attended 4 study visits: at baseline, 12 ± 1 wk, 24 ± 1 wk, and 36 ± 1 wk, operationalized as baseline, 12 wk, 24 wk, and 36 wk, respectively. Information on demographic characteristics, medication/supplement use, and history of acute pain episode frequency and severity were collected via questionnaires at baseline. Study participants were provided with 1 bottle of study supplements (100 capsules) after blood collection at baseline and at 24 wk.

At each study visit, a 3-h fasting venous blood specimen was either collected at BC Children’s Hospital or at a community-based laboratory (and transported at refrigeration temperatures of 0–4°C the same or next business day) and processed at BC Children’s Hospital BioBank. Participants were advised not to take their study supplement on the day of the blood collection.

A total of 10 mL of venous blood was collected in a 6 mL EDTA tube and a 4 mL serum tube (Becton, Dickinson and Company). For preparation of whole blood hemolysate, whole blood (0.3 mL) was removed from the EDTA tube and diluted 1/11 by adding 3.0 mL of a 1% ascorbic acid solution and subsequently incubated at 37°C for 30 min. The remaining volume of the EDTA tube was centrifuged at 1000 × *g* for 15 min at 4°C, and plasma and buffy coat were collected. The 4 mL serum tube was left at room temperature for ∼30 min (until clotted), centrifuged at 3000 × *g* for 15 min at 4°C, and serum was collected. All aliquots were stored at –80°C until further analysis.

RBC folate was measured using a microbiological assay with chloramphenicol-resistant *Lacticaseibacillus rhamnosus* (strain ATCC 7469) and folic acid as the standard following the method of Molloy and Scott [15]. RBC folate (nmol/L) was calculated as follows: RBC folate = [(whole blood hemolysate folate × 11) – serum folate (1-Hct/100)/Hct/100]. For individuals with a missing hematocrit measurement (*n* = 5), a fill value equivalent to the median hematocrit for the population (27%) was used [16].

Serum folate forms (nmol/L), including UMFA [limit of detection (LOD) = 0.27 nmol/L)], 5-methyletrahydrofolate (5-MTHF; LOD = 0.07 nmol/L), 5-formyltetrahydrofolate (5-FoTHF; LOD = 0.52 nmol/L), 4-⍺-hydroxy-5-MTHF (LOD = 0.2 nmol/L), and folate catabolites acetamidobenzoylglutamate (LOD = 0.13 nmol/L) and para-aminobenzoylglutamate (LOD = 0.08 nmol/L), were measured using liquid chromatography tandem mass spectrometry (LC-MS/MS). Interassay variability of each folate form was determined using internal quality control samples analyzed in duplicate; interassay coefficients of variation (CVs) ranged from 4% to 11% for serum folate forms and was 6% to 12% for plasma UMFA concentrations. The detailed description of quantification methods has been published [17,18]. Total serum folate (nmol/L) was calculated from the sum of UMFA, 5-MTHF, 5-FoTHF, and 4-⍺-hydroxy-5-MTHF concentrations. Total serum folate concentrations were used in the calculation of RBC folate concentrations detailed above. Reduced serum folate (nmol/L) was calculated from the sum of 5-MTHF, 5-FoTHF, and 4-⍺-hydroxy-5-MTHF concentrations. The WHO thresholds for folate deficiency were used: RBC folate <227 nmol/L and serum folate <7 nmol/L [19].

#### Post-hoc amendments to biological sample processing

In our published protocol, we specified that we would determine serum folate concentrations using a microbiological assay and RBC folate using LC-MS/MS. However, serum folate was quantified by LC-MS/MS to detect all serum folate forms, and RBC folate concentrations were quantified by the microbiological assay in keeping with widely accepted recommendations from the roundtable to inform folate assessment in the US National Health and Nutrition Examination Survey [20].

One-carbon metabolites including plasma total homocysteine (LOD = 0.1 μmol/L), SAH (LOD = 0.25 nmol/L), and SAM (LOD = 2.5 nmol/L) were measured using HPLC tandem mass spectrometry [21,22]. For the determination of vitamin B_12_ status, plasma MMA (LOD = 0.03 μmol/L) was measured using gas chromatography tandem mass spectrometry [18]. Plasma vitamin B_12_ was measured using an automated immunoassay analyzer (Architect i1000, Abbott Labs). The assay range was 61.2–1475.6 pmol/L. Results below the assay threshold were obtained by enrichment with low standard and results above the threshold were obtained by dilution. The interassay CV was 3% for MMA, 4% for total homocysteine, 5% for SAM, 7% for SAH, and 6% for vitamin B_12_.

Complete blood counts (CBC) and serum creatinine measures were prospectively collected from clinical bloodwork completed in tandem with research blood work. CBCs were performed using an automated hematology analyzer (Sysmex, Sysmex Corp.) and serum creatinine measures were performed using enzymatic methods at the BC Children’s and Women’s Hospital Laboratory. The CBC analysis provided measures of hemoglobin (g/L), hematocrit (%), mean corpuscular hemoglobin [mean corpuscular volume (MCV); fL], and counts of reticulocytes (×10^9^/L), platelets (×10^9^/L), and neutrophils (×10^9^/L). Megaloblastic changes were defined as an increase in MCV >3 fL and a reticulocyte count <100 × 10^9^/L, and/or unexplained neutropenia (neutrophils <1.5 × 10^9^/L) and thrombocytopenia (platelets <100 × 10^9^/L).

Genomic DNA was extracted from buffy coat using the QIAamp Blood Kit (QIAGEN). Genotyping of the *MTHFR* (677 C>T, rs1801133) and *DHFR* (rs70991108, 19 base-pair deletion/insertion) variants, which was completed because of the known influence of genetic polymorphisms of these enzymes on folate metabolism [23], was conducted by real-time PCR (7500 Real-Time PCR System; Applied Biosystems) using commercial TaqMan SNP Genotyping Assays (catalog number 4351379; Applied Biosystems).

Dietary folate equivalent intake was collected and analyzed using 24-h dietary recalls from the Automated Self-Administered 24-h Dietary Assessment Tool-Canada 2018 version. Participants were asked to complete 3 non-consecutive 24-h dietary recalls (at baseline, 12 wk, and 24 wk) and intake was averaged across recalls. For those <13 y of age, parents were encouraged to complete the dietary recall with their child, and children >13 y were encouraged to complete the dietary recall independently with parental assistance as required.

Information on all adverse events, including acute pain episodes, hospitalizations, and RBC transfusions, was recorded at the end of each 12-wk study period. All serious adverse events were assessed by the qualified medical investigator (the relevant physician) as either related, unrelated, or unknown relation to the study supplement and this assessment was subsequently adjudicated by a 3-member Data and Safety Monitoring Board. No serious adverse events were determined to be related to the study supplement during this trial.

Adherence to supplementation was determined by capsule counts and participant-completed supplement diaries at wk 12 and 36. For those who did not return unused capsules after the second intervention period (*n* = 2), adherence was assumed to be the same as in the first period.

### Outcomes

The primary outcome was the difference in mean endline RBC folate concentrations (nmol/L) between treatments, as assessed by paired t-tests. Secondary outcomes included: differences in mean or median endline serum folate and 1-carbon metabolite (plasma total homocysteine, SAH, SAM, vitamin B_12_, and MMA) concentrations, and the frequency of clinical outcomes (the occurrence of megaloblastic changes and acute pain episodes) between treatments.

### Statistical analysis

A sample size of 14 participants per treatment order (total of 28) was estimated to be required to confirm that the RBC folate concentrations are at most 100 ng/mL (227 nmol/L; the noninferiority margin determined based on an estimate of clinical significance, as informed by the mean difference observed by Nguyen et al. [12]) lower during the placebo rather than the folic acid supplementation period, with a power of 80%, and a 2-sided alpha of 0.05. The sample size assumed a single group SD in RBC folate of 150 ng/mL (340 nmol/L) and a difference in means between groups of 30 ng/mL (68 nmol/L) based on the Nguyen et al. study [12].

Descriptive statistics were applied for participant demographic and biochemical outcomes. Given the minimal amount of missing data, which was determined to be missing at random, no statistical imputation methods were applied. Modified intention-to-treat analysis, in which participants who were randomized to treatment and received ≥1 dose of study treatment were retained in their original assigned groups, was completed for the primary analysis. First, confirmation that the washout period was long enough to rule out a carry-over effect was conducted using a preliminary test in which the sum of the RBC folate concentrations at treatment endlines (weeks 12 and 36) was calculated for each subject and compared across the 2 sequence groups by an independent sample *t*-test using the formula described by Wellek and Blettner [24]. Subsequently, paired t-tests were used to assess the difference in mean endline RBC serum folate concentrations (at weeks 12 and 36) between treatments. The bounds of the 95% confidence interval (CI) around the difference in mean endline RBC folate concentrations (modified intention-to-treat) were then compared against the noninferiority margin (227 nmol/L). Ad hoc per-protocol analyses were also conducted as secondary analyses, which included the exclusion of individuals who had an RBC transfusion during the intervention period to limit the influence of exogenous folate from donor blood [25] and including only participants who were ≥70% adherent to supplementation and had completed the trial.

Additional ad hoc sensitivity analyses were performed to evaluate the robustness of the results. Linear mixed-effect models were used for the outcomes of endline RBC folate concentrations, controlling for treatment sequence and baseline RBC folate concentrations, and the change in RBC folate from baseline to endline, controlling for treatment sequence. We opted not to complete sensitivity analyses based on *MTHFR* or *DHFR* genotypes, as no participants had the homozygous (TT) *MTHFR* genotype and only 2 participants had the *DHFR* 19 bp deletion/deletion genotype (Supplemental Table 1).

For secondary outcomes, paired t-tests were also applied to assess for differences in mean endline concentrations of normally distributed data (serum folate, hemoglobin, hematocrit, MCV, and total homocysteine, SAH, and SAM concentrations) between treatments, and Wilcoxon matched-pairs signed-rank tests were applied to assess for differences in median endline concentrations of non-normally distributed data (plasma vitamin B_12_ and MMA concentrations, and % UMFA as a proportion of total serum folate) between treatments.

McNemar’s test was applied to determine if there were differences in the number of participants who had: deficient RBC folate concentrations, ‘detectable’ levels of serum UMFA (based on the LOD of the assay; >0.27 nmol/L), and acute pain episodes, megaloblastic changes, and RBC transfusions after each treatment period. Mann–Whitney *U* tests were applied to assess for differences in median folate intake between treatment groups.

Statistical analyses were performed using Stata BE/18 (Stata Corp). Two-sided *P* values <0.05 indicated statistical significance.

## Results

**FIGURE 1:**
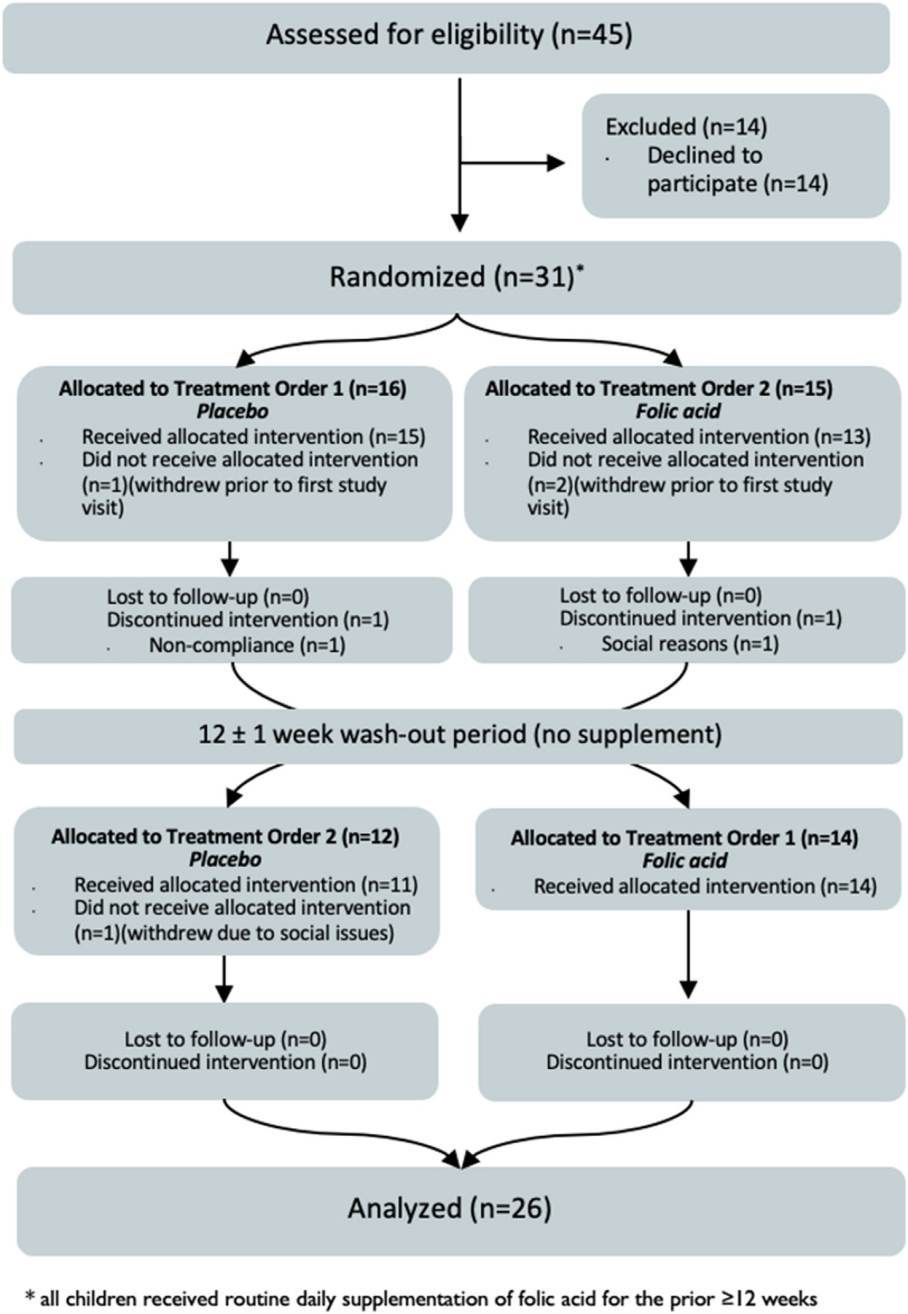
CONSORT flow diagram.

Between 14 October, 2020 and 31 October, 2022, a total of 31 individuals with SCD were randomized (1:1 with block sizes of 4) to either receive supplementation with folic acid then placebo or supplementation with placebo then folic acid (Figure 1). Overall, *n* = 28 individuals completed baseline bloodwork, *n* = 26 completed the first study intervention, and *n*=25 completed both interventions and had paired data that were eligible for the primary analysis (Figure 1).

Participants had a median (IQR) age of 10 (8–14) y. Most individuals (94%) had the homozygous hemoglobin SS genotype, and 6% had the hemoglobin Sβ^0^-thalassemia genotype. A total of 29 (94%) individuals were prescribed daily hydroxyurea, with a mean (±SD) dose of 21.1 ± 3.9 mg/kg/d. Additional participant characteristics are presented (Table 1). The mean (±SD) duration of intervention periods was 85 ± 7 d. Mean (±SD) adherence to study supplementation was 87% ± 16%. At the time of manufacture, only the folic acid supplements were confirmed to contain folic acid, with a measured content of 1.10 mg folic acid per capsule.

**TABLE 1:** Baseline characteristics, clinical indices, and biochemical indices of enrolled children with sickle cell disease^1^.

Characteristic	Treatment order		Total (*n* = 31)
	Placebo-folic acid (*n* = 16)	Folic acid-placebo (*n* = 15)	
Age (y), median (IQR)	10 (8–14)	10 (8–14)	10 (8–14)
Sex, *n* (%)			
Female	10 (62)	7 (47)	17 (55)
Male	6 (38)	8 (53)	14 (45)
SCD genotype, *n* (%)			
HbSS	15 (94)	14 (93)	29 (94)
HbSβ^0^-thalassemia	1 (6)	1 (7)	2 (6)
Region of ancestry^2^, *n* (%)			
Africa	12 (80)	13 (93)	25 (86)
Caribbean	2 (13)	0 (0)	2 (7)
Oceania	1 (7)	1 (7)	2 (7)
Medications			
Hydroxyurea, yes, *n* (%)	14 (88)	15 (100)	29 (94)
Dose (mg/kg), mean (SD)	21.2 ± 4.1	22.9 ± 3.7	22.1 ± 3.9
Prophylactic antibiotics, yes, *n* (%)	15 (94)	13 (87)	28 (90)
Folic acid supplementation, yes, *n* (%)	16 (100)	15 (100)	31 (100)
Reported adherence over last 7 days, median (range)^2^, *n* (%)	100 (0–100)	93 (57–100)	100 (0–100)
Reported adherence ≥ 5 days/wk^2^, *n* (%)	14 (93)	12 (86)	26 (90)

Retained participants	Placebo-folic acid (*n* = 15)	Folic acid-placebo (*n* = 13)	Total (*n* = 28)

Clinical indices			
Reported acute pain crisis in last 3 mo, yes, *n* (%)	3 (20)	3 (23)	6 (21)
Pain managed at home, yes, *n* (%)	3 (20)	2 (15)	5 (18)
Pain managed in ED, yes, *n* (%)	0 (0)	1 (8)	1 (0.04)
Pain managed in-hospital ward, yes, *n* (%)	0 (0)	0 (0)	0 (0)
Red blood cell transfusion in last 3 mo, yes, *n* (%)	0 (0)	0 (0)	0 (0)
Megaloblastic changes in last 3 mo, yes^3^, *n* (%)	0 (0)	0 (0)	0 (0)
Biochemical indices			
Hematological			
Hemoglobin (g/L), mean (SD)	89.1 (14.6)	94.6 (13.6)	91.6 (14.2)
Hematocrit (%), mean (SD)	26.1 (3.9)	27.2 (3.6)	26.6 (3.7)
MCV, fL, mean (SD)	92.8 (11.2)	92.6 (12.2)	92.7 (11.4)
Reticulocytes, ×10^9^/L^4^, mean (SD)	203.1 (99.3)	182.2 (68.5)	193.8 (86.1)
Neutrophils, ×10^9^/L, median (IQR)	2.7 (2.2–3.7)	2.5 (2.0–3.3)	2.6 (2.1–3.7)
Platelets, ×10^9^/L, median (IQR)	289 (169–438)	198 (125–276)	248 (146–355)

Characteristic	Treatment order		Total
	Placebo-folic acid	Folic acid-placebo	
Folate forms			
RBC folate (nmol/L), mean (SD)	629.7 (291.1)	484.6 (186.3)	562.3 (254.6)
RBC folate <227 nmol/L, *n* (%)	0 (0)	1 (8)	1 (0.04)
Total serum folate (nmol/L^5^), mean (SD)	57.2 (19.0)	78.0 (31.4)	66.9 (27.1)
Total serum folate <7 nmol/L, *n* (%)	0 (0)	0 (0)	0 (0)
5-MTHF (nmol/L), mean (SD)	51.7 (17.2)	62.3 (20.3)	56.6 (19.1)
4-⍺-hydroxy-5-MTHF, nmol/L, median (IQR)	3.6 (2.5–4.8)	2.9 (2.3–3.4)	3.0 (2.3–4.6)
UMFA (nmol/L), median (IQR)	0.6 (0.0–1.1)	0.0 (0.0–0.6)	0.0 (0.0–0.9)
UMFA >0.27 nmol/L, *n* (%)	8 (53)	5 (38)	13 (46)
5-FoTHF >0.52 nmol/L, *n* (%)	0 (0)	0 (0)	0 (0)

Abbreviations: 5-FoTHF, 5-formyltetrahydrofolate; 5-MTHF, 5-methyltetrahydrofolate; ED, emergency department; Hb, hemoglobin; RBC, red blood cell; SCD, sickle cell disease; UMFA, unmetabolized folic acid.

1 Values are frequencies (%), unless otherwise indicated.

2 *n* = 29; 2 participants did not complete demographic questionnaire.

3 Megaloblastic changes were defined as: increase in mean corpuscular volume >3 fL and a reticulocyte count <100 × 10^9^/L, and/or unexplained neutropenia (platelets <100 × 10^9^/L) and thrombocytopenia (neutrophils <1.5 × 10^9^/L).

4 At baseline, *n* = 1 participant in the placebo-folic acid group did not have reticulocytes measured.

5 Total serum folate represents the sum of UMFA, 5-MTHF, 5-FoTHF, and 4-⍺-hydroxy-5-MTHF. Total serum folate was used in the calculation of RBC folate.

**FIGURE 2:**
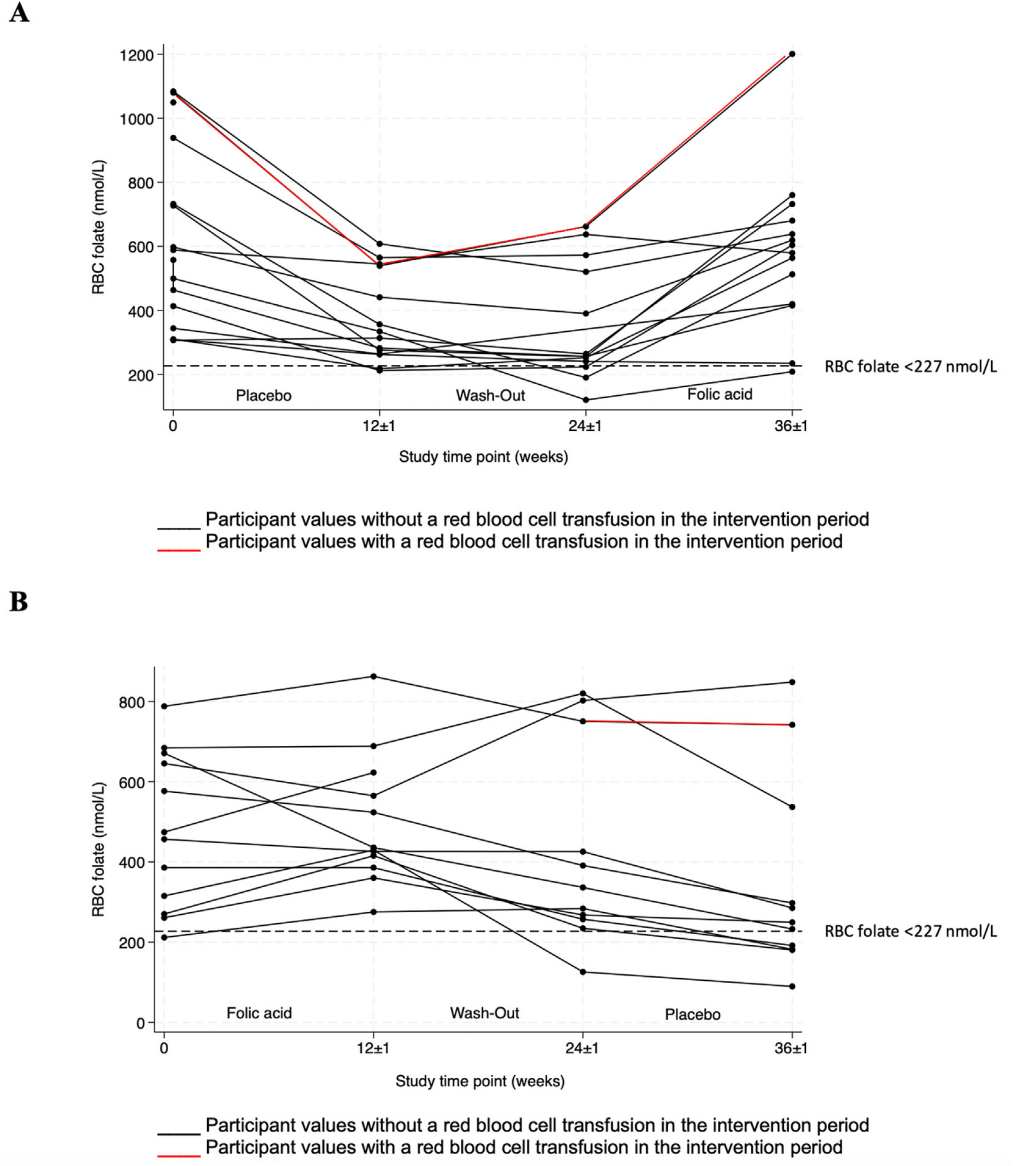
Red blood cell (RBC) folate trends by treatment order among children with sickle cell disease. (A) Red blood folate trends among participants who received placebo-folic acid treatment order (*n* = 14). (B) RBC folate trends among participants who received folic acid-placebo treatment order (*n* = 12).

Mean (SD) concentrations of RBC folate for all participants at baseline were 562 ± 254 nmol/L (Table 1). There was no significant difference in the number of participants who had RBC folate deficiency after each treatment (*P* = 0.059), although *n* = 6 participants were deficient after placebo but not after folic acid treatment, and *n* = 1 participant was deficient after folic acid treatment but not after placebo treatment (Figure 2).

**FIGURE 3:**
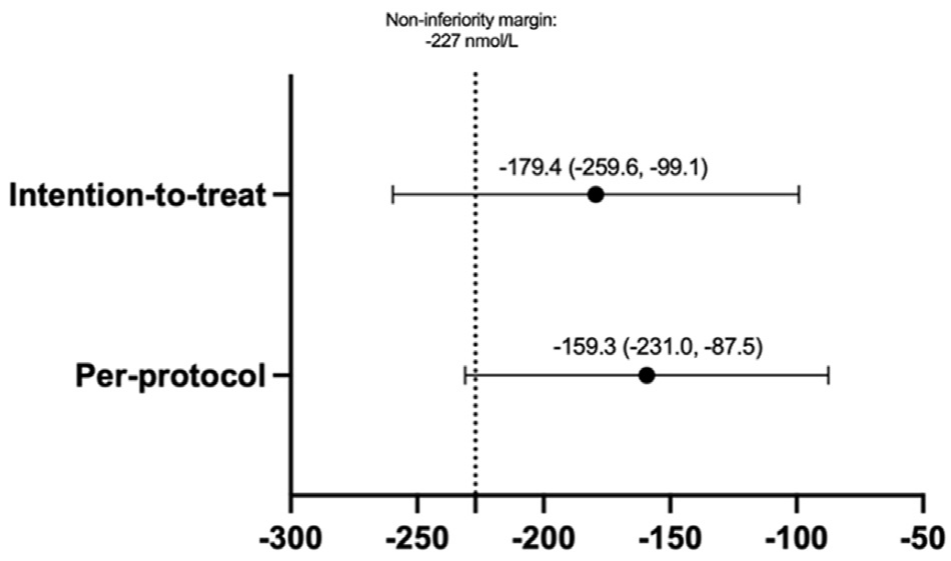
Mean difference (95% CI) in red blood cell folate concentration (nmol/L) between treatments (placebo compared with folic acid) using modified intention-to-treat and per-protocol analysis.

In modified intention-to-treat analyses, the mean (95% CI) difference in RBC folate concentrations across treatments (placebo compared with folic acid) was –179 (95% CI: –260, –99) nmol/L (*P* < 0.001; Table 2). In per-protocol analyses, excluding individuals who received an RBC transfusion and individuals who consumed <70% of capsules, the mean (95% CI) difference in RBC folate concentrations was –224 (95% CI: –336, –111) nmol/L (*P* < 0.001) (Table 2) and –183 (95% CI: –271, –96) nmol/L (*P* < 0.001) (Supplemental Table 2), respectively. Although the results were statistically significant, the lower bound of the CI was less than the a priori defined noninferiority margin of –227 nmol/L; hence, the results were equivocal regarding noninferiority of placebo in comparison with folic acid for the maintenance of RBC folate concentrations over 12 wk (Figure 3). Results from the sensitivity analyses were similar to results from the primary analysis, with a significant difference observed in RBC folate concentrations by treatment, but equivocal results regarding noninferiority of placebo in comparison with folic acid (Supplemental Table 2).

**TABLE 2:** Effect of supplemental folic acid or placebo on red blood cell folate, serum folate, hematological indices, clinical outcomes and 1-carbon metabolites among children with sickle cell disease^1^.

	Placebo (*n* = 25)	Folic acid (*n* = 25)	Paired mean difference (95% CI)	*P* value
Primary outcome				
RBC folate concentration (nmol/L)				
Intention-to-treat analysis	362.2 (190.6)	541.6 (214.4)	–179.4 (–259.6, –99.1)	<0.0001
Per-protocol analysis^2^	354.8 (191.0)	514.1 (168.1)	–159.3 (–231.0, –87.5)	<0.0001
Secondary outcomes				
Total serum folate (nmol/L^3^)	34.9 (16.1)	67.1 (48.4)	–32.2 (–52.5, –11.9)	0.0032
UMFA (nmol/L), median (range)	0.0 (0.0–12.5)	0.0 (0.0–151)	N/A	0.0566
% of total serum folate as UMFA, median (range)	0.0 (0.0–1.6)	0.0 (0.0–5.9)	N/A	0.0537
Reduced folate (nmol/L^4^)	34.2 (14.4)	57.2 (23.0)	–22.9 (–33.7, –12.2)	0.0002
Complete blood count measures				
Hemoglobin (g/L)	94.3 (16.3)	90.6 (10.4)	3.72 (–0.6, 8.0)	0.0854
Hematocrit (%)	27.9 (4.2)	27.0 (5.2)	0.9 (–0.6, 2.3)	0.2243
MCV, fL^2^	95.0 (12.5)	94.1 (11.7)	0.9 (–1.8, 3.6)	0.5057
Clinical outcomes, *n* (%)				
Acute pain crisis^5^, yes	2 (8%)	2 (8%)	N/A	1.0000
Managed at home, yes	1 (4%)	0 (0%)	N/A	1.0000
Managed in ED, yes	0 (0%)	0 (0%)	N/A	1.0000
Managed in-hospital ward, yes	1 (4%)	2 (8%)	N/A	1.0000
RBC transfusion, yes	2 (8%)	1 (4%)	N/A	1.0000
Megaloblastic changes^6^, yes	1 (4%)	0 (0%)	N/A	1.0000
One-carbon metabolites				
Vitamin B_12_ (pmol/L), median (IQR)	399.8 (307.6–466.6)	353.7 (302.7–516.7)	N/A	0.1183
MMA (nmol/L), median (IQR)	110 (90–140)	110 (100–140)	N/A	0.1377
Total homocysteine (μmol/L)	8.6 (3.3)	7.9 (3.4)	0.7 (–0.3, 1.7)	0.1777
SAH (nmol/L)	43.7 (16.2)	44.5 (15.9)	–0.8 (–8.4, 6.9)	0.8378
SAM (nmol/L)	107.6 (35.4)	107.2 (44.2)	0.4 (–18.1, 18.9)	0.9640

Abbreviations: ED, emergency department; MCV, mean corpuscular volume; MMA, methylmalonic acid; N/A, not applicable; RBC, red blood cell; SAH, *S*-adenosylhomocysteine; SAM, *S*-adenosylmethionine; UMFA, unmetabolized folic acid.

1 Values are mean (±SD), unless otherwise indicated.

2 Number of participants, *n* = 24.

3 Total serum folate represents the sum of UMFA, 5-MTHF, 5-FoTHF, and 4-⍺-hydroxy-5-MTHF concentrations. Total serum folate was used in the calculation of RBC folate.

4 Reduced folate represents the sum of 5-MTHF, 5-FoTHF, and 4-⍺-hydroxy-5-MTHF concentrations.

5 Acute pain crises defined as sudden onset of throbbing and continuous pain which occurred in 1 area of the body such as the back, joints, or arms/legs, or pain that moved throughout areas of the body.

6 Megaloblastic changes were defined as: increase in mean corpuscular volume >3 fL and a reticulocyte count <100 × 10^9^/L, and/or unexplained neutropenia (platelets <100 × 10^9^/L) and thrombocytopenia (neutrophils <1.5 × 10^9^/L).

### Secondary outcomes

**FIGURE 4:**
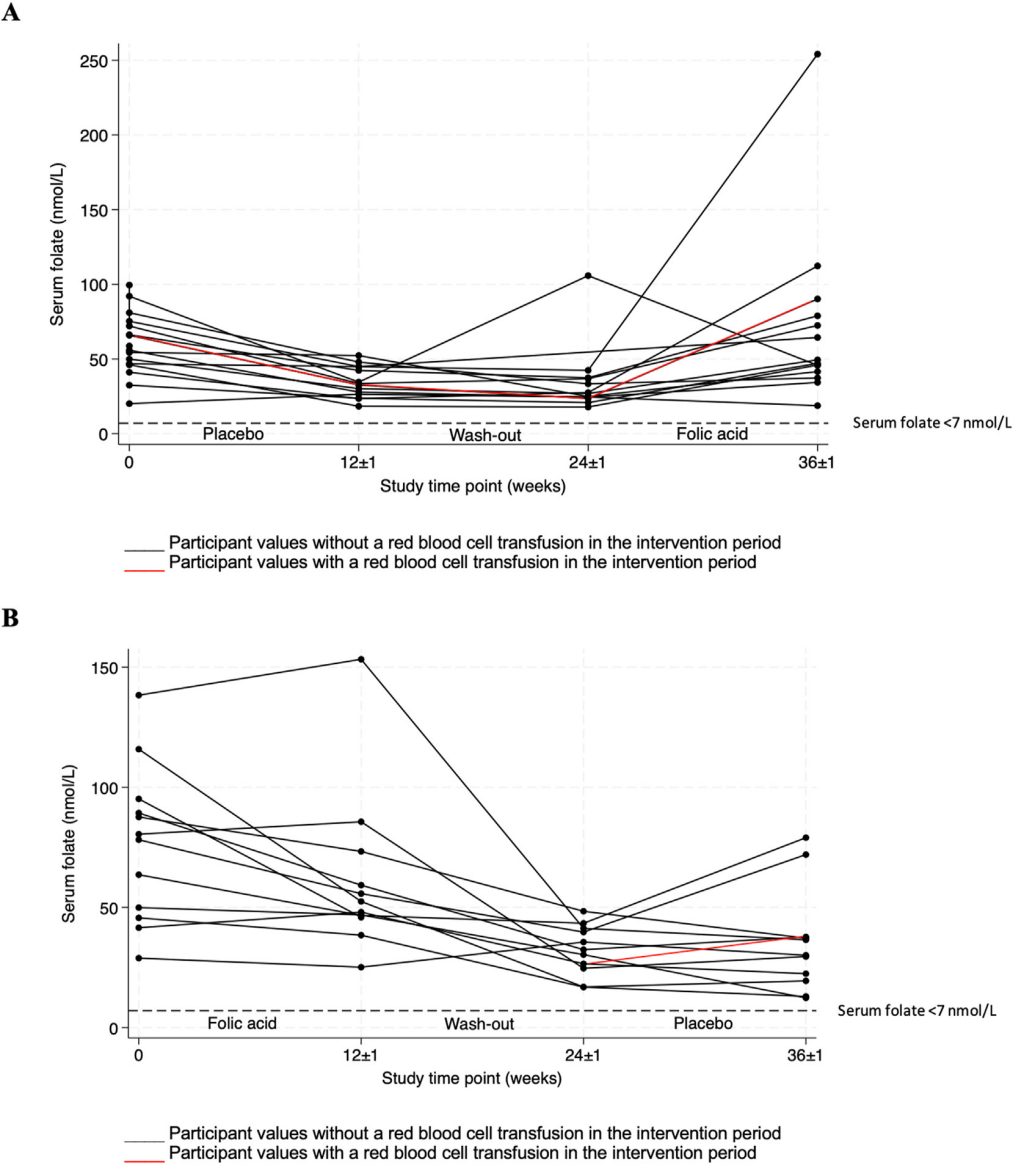
Serum folate trends by treatment order among children with sickle cell disease. (A) Serum folate trends among participants who received treatment order placebo-folic acid (*n* = 14). (B) Serum folate trends among participants who received treatment order folic acid-placebo (*n* = 12).

The mean (SD) concentrations of serum folate for all participants at baseline were 66.9 ± 27.1 nmol/L (Table 1). No participants presented with serum folate deficiency (<7 nmol/L) after either treatment (Figure 4). There was a significant difference in the proportion of individuals who had detectable levels of UMFA (>0.27 nmol/L) at the endline of treatment periods, as *n* = 2 individuals had detectable UMFA after both folic acid and placebo treatments, *n* = 1 had detectable levels only after the placebo period, *n* = 8 had detectable levels after just the folic acid period, and *n* = 14 individuals did not have detectable levels after either treatment (*P* = 0.020) (Supplemental Table 3).

One participant in the placebo-folic acid treatment order met our criteria to define a megaloblastic change at 12 wk (Supplemental Table 4). However, hematological measures had resolved without medical attention on standard repeat clinical blood work 1 wk later, and a serum folate concentration assessed at that time was 39.8 nmol/L (above the deficiency threshold). Thus, according to our protocol, the participant was deemed suitable to continue in subsequent interventions because of a lack of evidence that folate deficiency may have contributed to the observed biochemical changes.

There were no significant differences in plasma concentrations of vitamin B_12_, MMA, total homocysteine, SAH, or SAM, nor in mean differences in endline hemoglobin, hematocrit, or MCV between treatments (*P* > 0.05) (Table 2). There were no significant differences in the number of participants who had megaloblastic changes, acute pain episodes, or RBC transfusions during each treatment period (*P* > 0.05) (Table 2).

In total, 26 individuals completed ≥1 24-h dietary recall (*n* = 3 completed 1 recall, *n* = 2 completed 2 recalls, *n* = 21 completed 3 recalls). There was no significant difference in dietary folate intake between treatment groups [median (IQR) = 276 (254–397) μg dietary folate equivalents (DFE) per day in the placebo-folic acid treatment order (*n* = 14) compared with 325 (245–392) μg DFE per day in the folic-placebo treatment order (*n* = 12; *P* = 0.86)].

## Discussion

In this double-blind randomized cross-over trial among children with SCD, results were equivocal in confirming the noninferiority of placebo to 1 mg/d folic acid for the maintenance of RBC folate concentrations over 12 wk. Authors of the most recent Cochrane Review (2018) on folate supplementation in individuals with SCD suggested that because of the lack of supporting evidence for routine folic acid supplementation in SCD, there is a practical and ethical need for a randomized control trial to assess the efficacy of folic acid supplementation in this population [3]. Our study is now the first published randomized controlled trial in the post-folic acid fortification era, to our knowledge, to address this identified research gap.

In this study, our primary outcome was RBC folate concentrations, which are considered to be indicative of longer term folate status, for example, representing the previous 3–4 mo in healthy RBCs, whereas serum folate reflects recent status or dietary intake [26]. Additionally, RBC folate concentrations were deemed the more appropriate primary outcome as other clinical outcomes, such as megaloblastic changes (which are relatively rare) and acute pain episodes (which can be influenced by factors like disease severity and medical management), were considered more variable [27]. The suitability of RBC folate concentration as a marker of folate status in individuals with SCD, a hemolytic condition marked by abnormally shaped RBCs, however, has been previously questioned. Low RBC folate levels have been observed at higher rates in children with SCD in the United States receiving routine folic acid supplementation [28] compared with children in the general population without SCD [29]. This suggests that sickled red blood cells may have an altered folate-carrying capacity and/or that the increased demands of erythropoiesis in SCD lead to depletion of folate stores. In a study of children with SCD in Curaçao (2002), van der Dijs et al. demonstrated that mean (SD) RBC folate concentrations measured via immunochemistry analyzer were found to be ∼1.6 times higher in participants with hemoglobin SS (HbSS; *n* = 10) as compared with participants with hemoglobin SC (HbSC; *n* = 7) (1666 ± 273 compared with 1034 ± 504 nmol/L, respectively; *P* = 0.016) [30]. Authors of this study also observed an inverse relationship between RBC folate and hemoglobin concentrations [31]. Thus, on the basis of these results, it was concluded that the use of serum folate and homocysteine may be more accurate markers of folate status in SCD than RBC folate concentrations. However, this study was small (*n* = 17) and was conducted before the widespread use of hydroxyurea therapy for individuals with SCD.

In general, hemolyzed samples can influence the nutrient concentrations in plasma or serum as RBC contents are released into those fluids. As SCD is hemolytic anemia marked by chronic hemolysis, we suspect that hemolysis may have influenced the flux of folate from RBCs into plasma. In our study, although *n* = 7 individuals were found to have RBC folate concentrations below the threshold for deficiency (<227 nmol/L) during the study timeframe, no participant had serum folate concentrations below the threshold for deficiency (<7 nmol/L) and the median (range) concentration of serum folate after the placebo period was 33 (12–79) nmol/L. Further, there was no significant change in total homocysteine concentrations by treatment, likely because of participants maintaining sufficient serum folate concentrations.

In our study, RBC folate concentrations were measured using the microbiological assay, which is considered the reference standard for the measurement of RBC folate concentrations. In comparison, in a 2017 study conducted by Nguyen et al., where RBC folate concentrations were determined by direct chemiluminescent competitive immunoassay, participants with SCD who had folic acid discontinued for >80 days (*n* = 51) were found to have mean RBC folate concentrations of 609.9 ± 176.1 ng/mL (equivalent to ∼1382 ± 399 nmol/L) [12]. A recent study in 2020 conducted by Hunt et al. in Australia compared the RBC folate concentrations in *n* = 74 healthy non-pregnant females with use of both the microbiological assay (with 5-MTHF calibration) and an immunoassay and found that the immunoassay produced mean concentrations that were almost twice as high as the mean concentrations from the microbiological assay [32]. This resulted in a mean difference (95% CI) between the 2 assays of 793 (724–862) nmol/L [32]. These findings suggest that the methodology used to determine folate concentrations can have a notable impact on the results obtained, which poses obvious challenges for the comparison of folate results across studies. Despite the potential limitations associated with extrapolating RBC folate results obtained using immunoassay, we considered the findings from the Nguyen et al. study (United States population) to be the most suitable for establishing our noninferiority margin and informing our sample size calculation for our study population (Canada), based on the similarity of the study populations in terms of food folic acid fortification and medical management.

Similar to the findings from the landmark clinical trial of folic acid supplementation among children with SCD conducted by Rabb et al. in 1983 [10], we found no significant difference in hematological measures or clinical outcomes (specifically acute pain episodes or megaloblastic changes) when individuals were supplemented with folic acid in comparison with placebo. Our trial, however, was not powered to detect significant differences in clinical outcomes, as this would have required a substantially larger sample size which was not feasible given the number of children with SCD attending our hospital. Larger, multisite studies are needed to ascertain if folic acid supplementation directly translates to improved clinical outcomes in individuals with SCD.

The strengths of this study include its rigorous design as a randomized controlled cross-over trial and our assessment of several biomarkers of folate status, 1-carbon metabolites, and clinical outcomes. The use of a cross-over design, in which each child served as his/her own control, reduced the bias of confounding variables (for example, age, sex, and dietary intake) and provided a lower sample size requirement to determine a statistically significant treatment effect [24]. Unique to our study, we also included a measure of dietary folate intake. This is an important consideration given that children in Canada are estimated to consume a substantial amount of folate and folic acid through dietary sources in the era of mandatory folic acid fortification of refined grains [33].

We also acknowledge some potential limitations. Participants were instructed to fast for 3 h before blood collection; however, this may not have been feasible for some participants, especially young children. Recent intakes of folate-rich foods may have contributed to an increased serum folate concentration, and thus, could influence interpretation of these results. Before enrolment in the study, participants were already prescribed folic acid supplementation. We elected not to include a 12-wk washout period before the first study intervention, as the trial was already 36 wk (9 mo) in duration, and we sought to minimize participant burden as much as possible and to mitigate the risk of participant loss to follow up. However, the consumption of folic acid supplements before the start of the study may have had an influence on the results of our first study intervention period. Although we found no statistical evidence of a carry-over effect, we acknowledge that treatment carry-over effects can be difficult to rule out [34]. Lastly, our findings may not be generalizable to countries without similar folic acid food fortification practices, nor in settings with low rates of hydroxyurea use (as 94% of participants in our trial were prescribed daily hydroxyurea at baseline).

In conclusion, overall, results demonstrated that RBC folate concentrations were significantly lower during placebo supplementation in comparison with folic acid supplement but were equivocal in confirming the noninferiority of placebo to 1 mg/d folic acid for the maintenance of RBC folate concentrations in children with SCD. However, all participants were able to maintain adequate serum folate concentrations during the period of placebo supplementation. Furthermore, no alterations in 1-carbon metabolites were observed between treatments. Future in vitro research to examine the capacity of sickled RBCs to reflect folate status would be helpful in informing the assessment and interpretation of folate status in this population with abnormally shaped RBCs.

## Author contributions

The authors’ responsibilities were as follows – CDK, BAW, HM, AMD, JS, SV, JKW: conceptualization and design; BAW, HM, AA, JKW, CDK: data collection and project administration; BAW, CDK: writing - original draft; HM, AMD, JS, SV, AA, JKW: writing - review and editing; BAW, JS, CDK: formal analysis; CDK: supervision; BAW, CDK: had full access to all data and have verified the data reported in the manuscript; and all authors: read and approved the final manuscript.

## Data availability

The deidentified datasets used and/or analyzed during the current study will be provided to researchers who provide a methodologically sound proposal, after receipt of a signed data access agreement. These data will be available with publication. Statistical codes, informed consent forms, and other related documents are available from the corresponding author on reasonable request.

## Funding

This study was supported by project grants from the Canadian Institutes of Health Research (CIHR; #419565) and the Thrasher Research Fund (#14798). The funders of the study had no role in study design, data collection, data analysis, data interpretation, or in the writing of the report. BAW was supported by a CIHR Frederick Banting and Charles Best Canada Graduate Scholarship Doctoral Award (#434962). AMD is supported by an Investigator Grant from BC Children’s Hospital Research Institute. CDK is supported by a Canada Research Chair in Micronutrients and Human Health.

## Conflict of interest

The authors report no conflicts of interest.
