# Supplementary material for: Folic acid supplementation in children with sickle cell disease: a randomized double-blind noninferiority cross-over trial
Source: Am J Clin Nutr. 2025 Feb 5;121(4):910–20. doi: 10.1016/j.ajcnut.2025.02.001 (PMC12002192; doi:10.1016/j.ajcnut.2025.02.001)
Supplement: Multimedia component 1 [file mmc1.docx]

**Supplemental Online Content**

**Supplemental Table 1**. Genetic polymorphisms and one-carbon metabolism-related biochemical indices of enrolled children with sickle cell disease

**Supplemental Table 2**. Sensitivity analyses of the effect of supplemental folic acid or placebo on red blood cell folate (nmol/L) among children with sickle cell disease

**Supplemental Table 3.** Unadjusted folate and one-carbon-related metabolite concentrations among children with sickle cell disease, by treatment order, at study timepoints

**Supplemental Table 4.** Unadjusted hematological markers and clinical outcomes among children with sickle cell disease, by treatment order, at study timepoints

Supplemental Table 1: Genetic polymorphisms and one-carbon metabolism-related biochemical indices of enrolled children with sickle cell disease^1^

| Characteristic | Treatment order | | | |  |
| --- | --- | --- | --- | --- | --- |
| Retained participants | Placebo-folic acid (n=15) | | Folic acid-placebo (n=13) | | Total  (n=28) |
| Biochemical indices |  | |  | |  |
| *MTHFR* C677T genotype |  |  | |  | |
| CC | 11 (73%) | 10 (77%) | | 21 (75%) | |
| CT | 4 (27%) | 3 (23%) | | 7 (25%) | |
| TT | 0 (0%) | 0 (0%) | | 0 (0%) | |
| *DHFR* genotype |  |  | |  | |
| 19 bp (ins/ins) | 7 (47) | 8 (62) | | 15 (54) | |
| 19 bp (ins/del) | 6 (40) | 5 (38) | | 11(39) | |
| 19 bp (del/del) | 2 (13) | 0 (0) | | 2 (7) | |
| Folate catabolites |  | |  | |  |
| apABG, nmol/L | 0.6 (0.5, 0.7) | | 0.6 (0.5, 0.8) | | 0.6 (0.5, 0.7) |
| pABG, nmol/L | 2.5 (1.4, 4.4) | | 2.9 (1.3, 6.3) | | 2.7 (1.3, 4.4) |
| One-carbon metabolism related metabolites | | | | | |
| Vitamin B12, pmol/L | 421 (268, 474) | | 355 (305, 389) | | 364 (299, 468) |
| Vitamin B12 <150 pmol/L^2^ | 0 (0%) | | 1 (8%) | | 1 (0.04%) |
| Methylmalonic acid, nmol/L | 130 (90, 180) | | 100 (100, 110) | | 115 (95, 175) |
| Methylmalonic acid >370 nmol/L^3^ | 0 (0%) | | 0 (0%) | | 0 (0%) |
| Total homocysteine, µmol/L | 6.7 (5.7, 8.1) | | 6.3 (5.0, 6.9) | | 6.4 (5.6, 7.4) |
| Total homocysteine >15 µmol/L^4^ | 0 (0%) | | 1 (8%) | | 0 (0.04%) |
| SAH, nmol/L | 34 (21, 42) | | 26 (21, 48) | | 33 (21, 45) |
| SAM, nmol/L | 115 (105, 150) | | 135 (119, 141) | | 131 (106, 142) |

^1^ Values are median (IQR), mean (±SD), or frequencies

^2^ Vitamin B12 deficiency was defined as plasma vitamin B12 <150 pmol/L(32)

^3^ Elevated MMA was defined as plasma MMA >370 nmol/L(33)

^4^ Hyperhomocysteinemia was defined as plasma total homocysteine >15 µmol/L(34)

apABG, acetamidobenzoylglutamate; pABG, para-aminobenzoylglutamate; bp, base pair; ins, insertion; del, deletion; SAH, *S*-adenosylhomocysteine; SAM, *S*-adenosylmethionine

Supplemental Table 2: Sensitivity analyses of the effect of supplemental folic acid or placebo on red blood cell folate (nmol/L) among children with sickle cell disease

| Per-protocol analyses^1^ | | | | | |
| --- | --- | --- | --- | --- | --- |
|  | Placebo (n=25) | Folic acid  (n=25) | Paired mean difference (95% CI) | | p value |
| Model 1: Unadjusted endline RBC folate concentrations (nmol/L) | 353.6 (194.2) | 536.8 (221.8) | -183.1  (-270.7, -95.6) | | <0.0001 |
| Adjusted mixed effects linear regression analyses^2^ | | | | | |
|  | β | 95% CI | | p value | |
| Model 2: Endline RBC folate adjusted for treatment sequence | -181.4 | -255.5, -107.3 | | <0.0001 | |
| Model 3: Endline RBC folate adjusted for treatment sequence and baseline RBC folate | -252.9 | -334.7, -171.1 | | <0.0001 | |
| Model 4: Change in RBC folate from baseline to endline adjusted for treatment sequence | -299.6 | -396.3, -202.9 | | <0.0001 | |

^1^ Per-protocol analysis where children who consumed <70% of capsules (equivalent to taking study supplement capsules <5 out of 7 days per week) were excluded. N=23

^2^ N=26

Supplemental Table 3: Unadjusted folate and one-carbon-related metabolite concentrations among children with sickle cell disease, by treatment order, at study timepoints

| Outcomes | Treatment order: placebo-folic acid | | | | Treatment order: folic acid-placebo | | | |
| --- | --- | --- | --- | --- | --- | --- | --- | --- |
|  | Baseline  (n=15) | 12 weeks  (n=14) | 24 weeks  (n=13) | 36 weeks  (n=14) | Baseline  (n=13) | 12 weeks  (n=12) | 24 weeks  (n=11) | 36 weeks  (n=11) |
| Folate forms | | | | | | | | |
| RBC folate, nmol/L | 588^a^  (344, 939) | 324^b^  (264, 539) | 257^b,c^  (241, 520) | 592^a,b^  (420, 680) | 474^e^  (315, 646) | 433^e^  (401-594) | 336^e^  (257, 751) | 250^e^  (182, 537) |
| <227 nmol/L | 0 (0%) | 2 (14%) | 3 (21%) | 1 (7%) | 1 (8%) | 0 (0%) | 1 (9%) | 4 (36%) |
| Total serum folate, nmol/L | 55.9^a^  (46.1, 72.1) | 33.1^b^  (26.4, 44.9) | 27.2^b,c^  (24.4, 37.4) | 48.3^a,d^  (41.6, 78.9) | 80.5^e^  (50.0, 95.2) | 50.3^e^  (46.3, 66.3) | 32.4^f^  (24.6, 41.3) | 30.1^f,g^  (19.4, 37.7) |
| <7 nmol/L | 0 (0%) | 0 (0%) | 0 (0%) | 0 (0%) | 0 (0%) | 0 (0%) | 0 (0%) | 0 (0%) |
| 5-MTHF, nmol/L | 54.2^a^  (42.3, 67.1) | 29.7^b^  (23.6, 42.3) | 23.2^b,c^  (22.0, 28.5) | 44.7^a,b,d^  (35.6, 68.6) | 61.0^e^  (47.0, 76.7) | 45.5^e^  (40.3, 57.0) | 28.9^f^  (23.0, 35.2) | 28.6^f^  (18.3, 35.5) |
| 4-⍺-hydroxy-5-MTHF, nmol/L | 3.6^a^  (2.5, 4.8) | 2.9^a^  (2.1, 3.7) | 3.0^a^  (1.8, 5.5) | 3.7^a^  (2.3, 5.0) | 2.9^e^  (2.3, 3.4) | 3.6^e^  (2.1, 4.8) | 2.7^e^  (2.0, 2.9) | 1.7^e^  (1.3, 4.2) |
| UMFA, nmol/L | 0.6^a^  (0.0, 1.1) | 0.0^b^  (0.0, 0.0) | 0.0^a,b^  (0.0, 0.0) | 0.0^a,b^  (0.0, 0.7) | 0.0^e^  (0.0, 0.6) | 0.7^e^  (0.0, 1.7) | 0.0^e^  (0.0, 0.0) | 0.0^e^  (0.0, 0.0) |
| >0.27 nmol/L | 8 (53%) | 1 (7%) | 3 (21%) | 4 (29%) | 5 (38%) | 7 (58%) | 1 (9%) | 2 (18%) |
| Folate catabolites | | | | | | | | |
| apABG, nmol/L | 0.6^a^  (0.5, 0.7) | 0.5^a^  (0.4, 0.6) | 0.5^a^  (0.4, 0.6) | 0.7^a^  (0.4, 0.8) | 0.6^e^  (0.5, 0.8) | 0.6^e^  (0.5, 0.7) | 0.5^f^  (0.4, 0.6) | 0.5^e^  (0.4, 0.7) |
| pABG, nmol/L | 2.5^a^  (1.4, 4.4) | 1.9^a^  (1.6, 2.4) | 1.2^a^  (0.9, 3.0) | 2.8^a^  (1.8, 4.6) | 2.9^e^  (1.3, 6.3) | 2.9^e^  (2.0, 6.9) | 2.0^e^  (0.9, 4.2) | 2.5^e^  (1.5, 8.4) |
| One-carbon-related metabolites | | | | | | | | |
| Vitamin B12, pmol/L | 421^a^  (268, 474) | 396^a^  (308, 444) | 321^a^  (296, 406) | 330^a^  (278, 517) | 355^e^  (305, 389) | 355^e^  (310, 504) | 465^e^  (279, 524) | 413^e^  (246, 555) |
| <150 pmol/L | 0 (0%) | 0 (0%) | 0 (0%) | 0 (0%) | 1 (8%) | 1 (8%) | 1 (9%) | 1 (9%) |
| Methylmalonic acid, nmol/L | 130^a^  (90, 180) | 115^a^  (100, 140) | 130^a^  (100, 160) | 135^a^  (110, 160) | 100^e^  (100, 110) | 110^e^  (100, 120) | 100^e^  (90, 110) | 90^e^  (90, 140) |
| >370 nmol/L | 0 (0%) | 0 (0%) | 1 (7%) | 0 (0%) | 0 (0%) | 1 (8%) | 1 (9%) | 1 (9%) |
| Total homocysteine, µmol/L | 6.7^a^  (5.7, 8.1) | 8.0^a^  (6.7, 9.5) | 8.4^a^  (7.2, 9.6) | 7.9^a^  (6.4, 9.7) | 6.3^e^  (5.0, 6.9) | 6.2^e^  (5.2, 9.6) | 6.5^e^  (5.7, 9.1) | 7.7^e^  (6.1, 11.3) |
| >15 µmol/L | 0 (0%) | 0 (0%) | 1 (7%) | 1 (7%) | 1 (8%) | 1 (8%) | 1 (9%) | 1 (9%) |
|  |  |  |  |  |  |  |  |  |
| Outcomes | Treatment order: placebo-folic acid | | | | Treatment order: folic acid-placebo | | | |
|  | Baseline  (n=15) | 12 weeks  (n=14) | 24 weeks  (n=13) | 36 weeks  (n=14) | Baseline  (n=13) | 12 weeks  (n=12) | 24 weeks  (n=11) | 36 weeks  (n=11) |
| Continued |  |  |  |  |  |  |  |  |
| SAH, nmol/L^2^ | 33.7^a^  (21.2,42.3) | 42.2^a^  (33.9, 50.5) | 36.3^a^  (33.0, 54.5) | 43.9^a^  (37.5, 58.6) | 25.8^e^  (21.3, 47.5) | 41.4^e^  (24.2, 55.3) | 28.4^e^  (21.1, 58.8) | 44.1^e^  (33.3, 55.1) |
| SAM, nmol/L^2^ | 115^a^  (105, 150) | 113^a^  (77, 133) | 113^a^  (98, 152) | 102^a^  (77, 124) | 135 ^e^  (119, 141) | 119^e^  (64, 142) | 135^e^  (85, 140) | 98^e^  (92, 120) |

^1^ Values are medians (IQR) or frequencies (%). Values with a different superscript letter in each row are statistically different, as determined by Kruskal Wallis H-tests (p<0.05; Bonferroni-corrected for multiple comparisons) (Letters a-d for treatment order placebo-folic acid; e-h for treatment order folic acid-placebo)

^2^At 24 weeks, n=1 in treatment order placebo-folic acid had a missing value due to insufficient plasma volume for analysis

Values with a different superscript letter in each row are statistically different, as determined by Kruskal-Wallis H tests (p<0.05; Bonferroni-corrected for multiple comparisons) (Letters a-d for treatment order placebo-folic acid; e-h for treatment order folic acid-placebo).

RBC, red blood cell; apABG, acetamidobenzoylglutamate; pABG, para-aminobenzoylglutamate; UMFA, unmetabolized folic acid; 5-MTHF, 5-methyltetrahydrofolate; SAH, *S*-adenosylhomocysteine; SAM, *S*-adenosylmethionine

Supplemental Table 4: Unadjusted hematological markers and clinical outcomes among children with sickle cell disease, by

treatment order, at study timepoints^1^

| Outcomes | Treatment order: placebo-folic acid | | | | Treatment order: folic acid-placebo | | | |
| --- | --- | --- | --- | --- | --- | --- | --- | --- |
|  | Baseline  (n=15) | 12 weeks  (n=14) | 24 weeks  (n=14) | 36 weeks  (n=14) | Baseline  (n=13) | 12 weeks  (n=12) | 24 weeks  (n=11) | 36 weeks  (n=11) |
| Hematological measures | | | | | | | | |
| Hemoglobin, g/L | 91^a^  (74, 96) | 94^a^  (81, 99) | 88^a^  (80, 95) | 91^a^  (79, 95) | 93^e^  (89, 102) | 92^e^  (83, 103) | 91^e^  (86, 104) | 95^e^  (86, 111) |
| Hematocrit, % | 26^a^  (24, 27) | 27^a^  (27, 29) | 26^a^  (25, 29) | 27^a^  (26, 30) | 27^e^  (25, 30) | 27^e^  (23, 30) | 28^e^  (25, 29) | 28^e^  (25, 31) |
| MCV, fL | 92^a^  (86, 100) | 94^a^  (85, 101) | 99^a^  (87, 101) | 93^a^  (90, 103) | 89^e^  (85, 100) | 96^e^  (85, 107) | 88^e^  (83, 106) | 94^e^  (82, 107) |
| Reticulocytes, x10^9^/L^2^ | 209^a^  (104, 259) | 179^a^  (99, 218) | 198^a^  (115, 247) | 193^a^  (157, 228) | 170^e^  (153, 217) | 178^e^  (145, 193) | 214^e^  (129, 220) | 210^e^  (139, 225) |
| Neutrophils, x10^9^/L | 2.7^a^  (2.2, 3.7) | 4.3^a^  (2.5, 5.3) | 3.9^a^  (2.3, 4.7) | 3.4^a^  (2.6, 4.5) | 2.5^e^  (2.0, 3.3) | 3.4^e^  (2.4, 4.3) | 2.8^e^  (2.3, 4.0) | 3.5^e^  (1.9, 5.0) |
| Platelets, x10^9^/L | 289^a^  (169, 438) | 289^a^  (245, 380) | 256^a^  (199, 432) | 260^a^  (204, 307) | 198^e^  (125, 276) | 221^e^  (123, 344) | 164^e^  (147, 316) | 285^e^  (188, 414) |
| Clinical outcomes | | | | | | | | |
| Acute pain crisis^3^, yes | 3 (20%) | 2 (14%) | 1 (7%) | 1 (7%) | 3 (23%) | 1 (8%) | 0 (0%) | 0 (0%) |
| Managed at home, yes | 3 (20%) | 1 (7%) | 0 (0%) | 0 (0%) | 2 (15%) | 0 (0%) | 0 (0%) | 0 (0%) |
| Managed in ED, yes | 0 (0%) | 0 (0%) | 0 (0%) | 0 (0%) | 1 (8%) | 0 (0%) | 0 (0%) | 0 (0%) |
| Managed in-hospital ward, yes | 0 (0%) | 1 (7%) | 1 (7%) | 1 (7%) | 0 (0%) | 1 (8%) | 0 (0%) | 0 (0%) |
| Red blood cell transfusion, yes | 0 (0%) | 1 (7%) | 1 (7%) | 1 (7%) | 0 (0%) | 0 (0%) | 0 (0%) | 1 (9%) |
| Megaloblastic changes^4^, yes | 0 (0%) | 1 (7%) | 0 (0%) | 0 (0%) | 0 (0%) | 0 (0%) | 0 (0%) | 0 (0%) |
| ^1^ Values are medians (IQR) or frequencies (%). Values with a different superscript letter in each row are statistically different, as determined by Kruskal Wallis H-tests (p<0.05; Bonferroni-corrected for multiple comparisons) (Letters a-d for treatment order placebo-folic acid; e-h for treatment order folic acid-placebo)  ^2^ At baseline, n=1 participant in placebo-folic acid group did not have reticulocytes measured, and at 36 weeks, n=1 participant in both the placebo-folic acid and in folic acid-placebo groups did not have reticulocytes measured  ^3^ Acute pain crises defined as sudden onset of throbbing and continuous pain which occurred in one area of the body such as the back, joints, or arms/legs, or pain that moved throughout areas of the body  ^4^ Megaloblastic changes were defined as: increase in mean corpuscular volume >3 fL and a reticulocyte count <100 x 10^9^/L, and/or unexplained neutropenia (platelets <100 × 10^9^/L) and thrombocytopenia (neutrophils <1.5 x 10^9^/L)  IQR, interquartile range; MCV, mean corpuscular volume; ED, Emergency Department | | | | | | | | |
